# Identifying Factors Influencing the Implementation of Early Intervention Services for Psychosis in Quebec, Canada: A Qualitative Study of Health Care Providers' Perspectives

**DOI:** 10.1111/eip.70104

**Published:** 2025-10-02

**Authors:** Bastian Bertulies‐Esposito, Paula Pires de Oliveira Padilha, Ruben Valle, Srividya N. Iyer, Amal Abdel‐Baki

**Affiliations:** ^1^ Department of Psychiatry Université de Montréal Montreal Quebec Canada; ^2^ Centre intégré universitaire de santé et de services sociaux du Centre‐Sud‐de‐l'île‐de‐Montréal Montreal Canada; ^3^ Centre de recherche du Centre Hospitalier de l'Université de Montréal Montreal Canada; ^4^ Department of Psychiatry McGill University Montreal Canada; ^5^ Douglas Mental Health Institute Montreal Canada; ^6^ Centre hospitalier de l'Université de Montréal Montreal Canada

**Keywords:** early intervention services, first episode psychosis, mental health services, service implementation

## Abstract

**Introduction:**

Early intervention services for psychosis are clinically and cost‐effective. Despite the availability of national and international guidelines and well‐identified essential components of high‐quality services, the implementation of early intervention services varies greatly. However, factors underpinning this are poorly understood and infrequently studied. In Quebec, the provision of earmarked funding and political support since 2017 has resulted in widespread dissemination of early intervention, which presented a valuable context to examine what shapes and influences the implementation of early intervention for psychosis.

**Methods:**

An online survey was sent to leaders of all early intervention programmes (*n* = 33) in Quebec to assess service organisation and delivery. The survey's qualitative component included open‐ended questions about factors impacting implementation, which are the focus of this report. Deductive and inductive thematic analysis was conducted through multiple iterations to reach consensus.

**Results:**

Twenty‐seven programmes responded to the questionnaire. Factors influencing implementation were separated into eight themes: human resources, workload, finances, physical resources, training, service delivery, service users and relationship with management. Every theme and subtheme was represented as a potential barrier or facilitator, with work atmosphere, quality of the clinic's premises, and management buy‐in of early intervention more frequently noted as facilitators.

**Conclusion:**

Factors at the organisational, service and staff levels affect the implementation of early psychosis programmes. Despite political support and increased funding, insufficient funding and its consequences, along with limited implementation supports, remain important barriers to successful implementation. Rapid learning health systems can provide effective feedback to programmes to identify strategies to overcome identified barriers and enhance understanding of interactions between the identified factors. Lived experiences perspectives should also be included in future implementation research.

## 1 Introduction

Early intervention services for psychosis (EIP) involve multidisciplinary teams that provide specialised care during the critical first 2–5 year period following the onset of psychosis (Birchwood et al. 1998) and have been shown to improve clinical and functional outcomes (Correll et al. 2018; Puntis et al. 2020) and be cost‐effective (Shields et al. 2022). Some of the essential components, which are described in national and international guidelines (Addington et al. 2018, 2013; Early Psychosis Guidelines Writing Group and EPPIC National Support Program 2016; Mascayano et al. 2019), include early detection, timely access, continuity of care, intensive case management, low dose of antipsychotics and low patient‐to‐clinician ratios to allow for appropriate intensity of services and outreach interventions.

However, the existence of guidelines alone has been shown to be insufficient to guarantee adherence to key components of EIP, which results in evidence‐practice gaps and heterogeneity across programmes and providers, both of which can contribute to difficulties in obtaining optimal outcomes (Csillag et al. 2016, 2018). An intervention or treatment will not be effective if it is not properly implemented. Hence, programme monitoring and continuous improvement are key to achieving desired clinical and service outcomes by increasing fidelity to the model.

In parallel with its dissemination in Australia, United Kingdom, Singapore, Denmark and other parts of Canada, which started in the 1980s (McGorry 1993), clinicians implemented EIP in Quebec's publicly funded healthcare system over a 30‐year period without provincial standards, guidelines or dedicated funding. In Quebec, all psychiatric care is free of charge for service users, including interdisciplinary interventions, such as EIP. However, funding varies between healthcare organisations, and budgets are decided by local managers, resulting in piecemeal implementation of services. To support the burgeoning community of clinicians and researchers in EIP, the *Association québécoise des programmes pour premiers épisodes psychotiques* (Quebec Association for early intervention in psychosis programmes; AQPPEP) was created in 2004 (L'Heureux et al. 2007). The association's scientific and public education activities, and lobbying efforts eventually led to the provincial government announcing measures to promote widespread implementation of EIP in 2017: the publication of standards (the *Cadre de référence: Programmes d'intervention pour premiers épisodes psychotiques*, hereafter referred to as ‘the *Cadre*’; Ministère de la santé et des services sociaux 2022), specific and protected funding and a specialist counsellor offering on‐site implementation support.

Eighty‐eight percent of Quebec's population had access to EIP in 2020 (compared to 46% in 2016). Our prior work showed that most Quebec EIP for psychosis offered high‐quality clinical services, in line with the Cadre's recommendations (Bertulies‐Esposito et al. 2022). However, some implementation challenges were also highlighted: delays for initial contact and assessment, difficulties maintaining recommended patient‐to‐case manager ratios; high workload and insufficient staffing (Bertulies‐Esposito et al. 2022).

A recent systematic review has shown that factors influencing implementation in EIP were related to systems, services and staff, with insufficient funding cited as the most prevalent barrier. Furthermore, staff's belief in the philosophy of early intervention was identified as an important facilitator of its successful implementation (Darker et al. 2023). This study expands on previous work by identifying both facilitators and barriers during the process of widespread implementation of EIP for psychosis, which was catalysed by dedicated funding across 33 sites in Quebec, Canada. This will provide insight to potential avenues to improve the implementation and dissemination process, and it will be an opportunity to look to contextual factors that may mediate this process.

## 2 Methods

### 2.1 Study Design

This qualitative study is part of a larger cross‐sectional descriptive study involving an online survey, which was described in previous work (Bertulies‐Esposito et al. 2022). The qualitative part of the survey consisted of 12 open‐ended questions. Topics included institutional support, team cohesion and morale, workload, integration of new practices, human, financial and physical resources and training. These topics were identified in studies on factors influencing service implementation in various evidence‐based behavioural health programmes, such as assertive community treatment (Mancini et al. 2009; Moser et al. 2004), illness recovery management (McHugo et al. 2007; Whitley et al. 2009) and substance use disorder treatment (Lundgren et al. 2012). All participants provided written consent to publication of the data as reported in this article. Ethics approval was obtained from the *Centre de recherche du Centre hospitalier de l'Université de Montréal* (project number 19.342).

### 2.2 Data Analysis

Data was analysed through thematic analysis (Braun and Clarke 2006, 2022). In this deductive and inductive process, two independent raters (P.P. and R.V.) used initial codes originating from the survey questions, and additional codes were generated inductively by analysing the data. These codes were subsequently categorised into themes and subthemes, which were then reviewed with B.B.‐E., and two other coding iterations were generated and reviewed by P.P., R.V. and B.B.‐E. Finally, all authors, including both senior authors (A.A.B. and S.I.), met for a team analysis to review the complete dataset and analysis, generating further themes and subthemes. Thereafter, data was re‐coded by P.P., R.V., and B.B.‐E. to ensure the appropriateness of these themes, with consensus reached after discussion. Finally, another team meeting led to the reorganisation and renaming of themes to better reflect the data, and consensus was reached between all authors.

The valence of each statement (i.e., positive, negative, or neutral content of each statement) with regards to the final themes was rated by B.B.‐E. and reviewed by P.P., and consensus was reached through team discussion. Descriptive statistics were used to conduct secondary analyses, such as trends in segment valence for themes and subthemes.

We adopted a reflexive approach when considering the positionality and perspectives of the research team. We acknowledged that all authors practice and are immersed in the medical model of EIP. It is also noteworthy that the senior authors are experts in the field of EIP in which they were involved both as clinicians and researchers. A.A.B. is also involved in management. These considerations may influence our understanding of factors influencing programme implementation from a medical and leadership perspective. However, the team included more junior clinician‐researchers with experience in healthcare settings outside of North America or Western, educated, industrialised, rich and democratic (WEIRD) countries, who may have noticed certain components of the data that the other authors could have missed due to their own experience and positionality. This may have contributed somewhat to mitigating the risk of bias, along with regular team meetings to discuss interpretations and coding, ensuring all viewpoints were considered and taken into account at each step of the data analysis.

## 3 Results

Of the 33 EIP that were invited to complete the survey, 28 responded to the whole questionnaire (one of these did not complete the open‐ended questions regarding programme implementation). One small programme declined to participate due to time constraints, and four did not respond. The programmes' lead psychiatrist (6/27; three were also programme directors) or clinical team leader (21/27) responded to the survey. Programmes were categorised on urbanicity (*n* = 13 urban programmes, *n* = 7 in middle‐sized cities, and *n* = 7 in rural locations), academic affiliation (*n* = 17 academic programmes and *n* = 10 non‐academic ones), and their duration of existence (*n* = 17 programmes founded prior to 2017 and *n* = 10 programmes founded afterwards). A total of 280 statements were recorded, composed of 554 coded segments. Of these, 172 had a positive valence, 325 a negative valence, 33 contained positive and negative elements and 24 were neutral.

**FIGURE 1:**
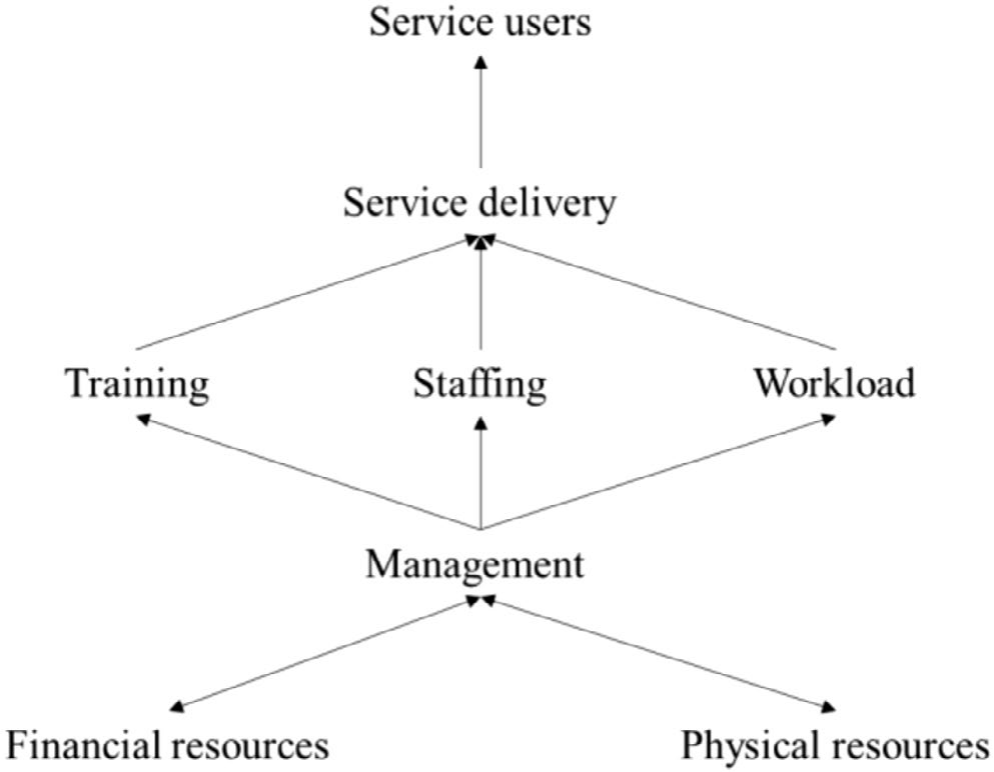
Schematic representation of identified themes of factors influencing implementation.

Figure 1 shows the eight main themes regarding factors influencing the implementation of EIP that we identified: human resources, workload, finances, physical resources, training, service delivery, service users and relationship with management. This last theme was the only one that was mostly considered a facilitator in implementation, whereas physical resources were equally noted as barriers and facilitators.

### 3.1 Human Resources

Availability of human resources was the most prevalent theme, with over 150 statements. Availability of clinical and administrative staff and turnover were the most cited barriers. Four subthemes were identified: quantity of clinical staff, turnover, administrative support and workplace atmosphere.

Insufficient staffing and lack of diverse or specifically needed disciplines among staff can affect the availability and delivery of interdisciplinary services in EIP, with consequences for staff and service users.

We would need […] an additional psychiatrist and two more case managers to make the program function properly. A psychologist attached to the program would also be an important addition. (Programme 22)

[We've had] difficulty doing outreach […] due to poor staffing. Improved staffing will allow us to fulfill our mandate. (Programme 9)

We do not have enough staff to increase our group offerings, to participate in research projects, but the 3 full‐time case managers have reduced overload and allow for better quality services. (Programme 1)

On the other hand, when EIP benefits from sufficient clinical staff, it is identified as a facilitator.

We used to be overloaded, but the recent increase in the number of staff has helped to rectify this. (Programme 9)

The addition of a full‐time worker allowed for a better distribution of the workload among the case managers. (Programme 1)

We have [team members from] all job titles, so we can offer multidisciplinary services. (Programme 14)

Having enough staff allows us to provide services that match the needs of patients. (Programme 18)

High staff turnover and difficulty in replacing staff on maternity or sick leave were seen as worrying, causing disruptions in team processes and work overload.

Given the turnover of case managers and the size of the team, it is difficult to standardize practices. (Programme 13)

A lot of staff turnover, so every time a new staff joins the [early intervention service], he/she needs to learn and master these best practices (Programme 28)

High‐quality administrative support was noted as key in reducing case manager workload, and its lack was considered a barrier to successful implementation and operation of EIP. Rural programmes tended to rate their administrative support higher than urban and middle‐sized ones.

Good administrative support in the psychiatric outpatient clinic, which facilitates clerical tasks such as sending mail/faxes, managing appointments. [This] allows case managers to focus more on patients. (Programme 6)

We no longer have dedicated administrative support for the clinic. [Clerical tasks, such as making] appointments, [noting] absences and [patient] arrival [at the clinic] are more difficult to manage (Programme 18)

The workplace atmosphere, team cohesion, clinicians' motivation for their work, ongoing development, their belief in the programme's model and the ability to instil optimism among staff were significant implementation facilitators. Also, out‐of‐office activities and social gatherings were highly regarded by several programme leaders as contributors to developing their team.

Our ability to work as a team and to seek support from colleagues when needed. Our ability to take things with humor. (Programme 5)

Solidarity, support between colleagues, co‐intervention, positive climate, everyone likes their job (they have chosen to be on the team), attached to clients. (Programme 14)

A common philosophy, commitment and mutual aid between workers, support from our manager.(Programme 20)

[There are several] positive elements: healthy and regular communication between members, Christmas dinner, ‘happy hour’. (Programme 1)

[There is a] culture of humor, de‐dramatization, [and] trial and error. (Programme 14)

[We have] young and dynamic case managers who believe in the program and who want to work with this clientele and their network. (Programme 3)

### 3.2 Workload

Work overload was strongly noted as an obstacle in implementing EIP. Higher staff caseloads, reluctance to admit new referrals due to insufficient staff, staff leaving and difficulties in replacing them, lack of administrative support and manager pressure to discharge patients were identified as possible factors that led to work overload or resulted from it.

[There is work] overload [, and] accumulation of complex cases. (Programme 24)

The successive entry of new referrals, patients who require multiple approaches. [The] departure of psychiatrists due to maternity leave overloads the psychiatrists of our program. [The addition of] a full‐time worker allowed for a better distribution of the workload among the case managers. (Programme 1)

[There are] limits to the number of admissions and follow‐ups because of a lack of clinical staff. There is also a lot of pressure from the management for quick discharges. (Programme 21)

### 3.3 Finances

Many statements reflected an underfunding of early intervention, which led to insufficient staffing and difficulties in appropriate service delivery.

Initially, we were told we had a budget for outreach which is not quite accurate. We have projects in mind, but we don't have the means to do them. (Programme 15)

[There is a] limited budget for therapeutic activities. Group activities and therapeutic material are largely financed by the hospital Foundation [philanthropy]. (Programme 20)

We had a housing program that was discontinued due to lack of funding. We would like to hire a peer worker but don't have funding for it. (Programme 25)

### 3.4 Physical Resources

Availability of physical and technological infrastructure was equally cited as barriers and facilitators to implementation. The main aspects highlighted were access to mobile phones, laptops, and car fleets, as well as adapted therapeutic premises. Though urban programmes tended to consider the quantity of available physical resources more positively than others, their quality and organisation were more often brought up as a barrier to seamless programme implementation and operation.

The addition of a laptop computer allows us to be more efficient in administrative tasks and to use it for therapeutic approaches, with clients, in the community. We have an adequate place for our team meetings and internet/network access in this room. (Programme 1)

[We] lack office space, but [there is a] new pavilion under construction. [There are] no windows in the offices: negative impact on the morale of some case managers. The premises are new, but cold and very institutional […] which makes the youth‐friendly approach more difficult. (Programme 13)

Potential safety risks for clinicians were also brought up.

[…The] clinic's mobile phones are shared among the case managers, which greatly complicates the work and does not ensure the safety of case managers during home visits. (Programme 6)

### 3.5 Training Needs

Continued education and training were identified as significant challenges, especially for programmes with high turnover rates. Gaps in specific training needs to meet the provincial standards were also highlighted. Several programmes referred to difficulties filling the specialist roles for case managers outlined in the Cadre (Quebec's provincial guidelines): substance use, family therapy, cognitive behavioural therapy for psychosis and vocational and educational support.

[There are] insufficient training [opportunities] and experienced clinicians in our specialist roles. (Programme 15)

We lack training [in] violence risk [assessment], cognitive behavioral therapy, motivational interviewing. (Programme 2)

[We] need more training for case managers on the specialist roles proposed by the standards (Programme 13)

Increased development of training opportunities through webinars or videoconferences was wished for by several programmes.

[We would appreciate] more training via videoconference because we are in a rural region. (Programme 7)

Trainings in different cities are sometimes difficult to reconcile with work and family [obligations]. [It is] preferable that they are less than an hour away or through videoconferencing. (Programme 15)

### 3.6 Service Delivery

Adequate service delivery, according to provincial guidelines, was hindered by inadequate staffing and funding, especially regarding outreach and intensity or duration of case management. Some programmes relied on other services to compensate for their inability to offer services to all FEP patients adequately, and group interventions were often underdeveloped or removed due to conflicting priorities and limited resources. Programmes located in middle‐sized cities or rural areas, as well as academic programmes, reported more positive aspects of using guidelines than other programme types.

[We] do not work in case management, [making] the workload more complex for psychiatrists. (Programme 16)

Our team members are all part‐time and overburdened. As a result, there is little energy left for community‐based interventions, outreach, and innovative group work with patients and their families. (Programme 22)

The case managers are competent and concerned about the clients' recovery. However, because of the number of requests and the needs of the clientele, we sometimes must make choices […] and reduce the group offer to respond to more urgent needs: housing, immigration, etc. (Programme 20)

On the other hand, having appropriate resources enabled programmes to deliver standard services.

Having enough [staff] allow us to provide services that match the needs of patients. (Programme 18)

Another feature mentioned by programmes was the importance of collaboration and communication with other services (e.g., community organisations, youth shelters, housing services, primary care, etc.) to deliver appropriate interventions and promote continuity of care:

We operate in a vast catchment area, [with] important needs for outreach. We use [case management/flexible assertive case management] teams for outreach needs. (Programme 27)

Standards for delivering care in early intervention matter. Tension was noted between creating services derived from gold standards versus adaptations to local contexts. In Quebec, a set of standards (Ministère de la santé et des services sociaux 2022) is in place to guide service development and to address practice gaps.

Some programmes deviated from standards to adapt to local circumstances, including seeking support from other general case management teams to offer timely services to youth with FEP.

[Faced with workload challenges, we] see some of the [FEP] referrals in another clinic where there are more clinicians, who are not FEP specialists, however. (Programme 16)

[To improve access to services, we] increased the clinicians' caseloads. [We] attempted to get places for our patients in the general clinic's groups, which did not always work. We also had support from other case management teams. (Programme 21)

We had to explain repeatedly the issues related to lack of human resources to meet the Cadre, [and] maintain standards for accessibility, reduced hospitalization length and waiting times for the outpatient services. (Programme 28)

### 3.7 Service Users

Despite clinical and psychosocial factors that increase intervention complexity, challenging teams in providing high‐quality care, some programme leaders noted a sense of accomplishment in developing creative solutions to complex situations. Conversely, this complexity and undesirable clinical outcomes were highlighted by several programmes.

The possibility for creativity in the delivery of services (services that are adapted to the young clientele). (Programme 25)

We enjoy working with this clientele despite [its] challenges. (Programme 4)

[We receive] too many clients and referrals, severe psychopathology. (Programme 22)

[We had] two suicides in a short time span. (Programme 28)

### 3.8 Relationship With Management

Flexibility of managers and their belief in the philosophy of early intervention in the management cadre were noted as influential factors in programme implementation, as were perceptions about management‐leadership. Despite most statements reporting positively on management‐leadership, urban and non‐academic programmes expressed more negative views about the leadership of the management.

Managers who believe in the [early intervention] program and recognize the unique value of early intervention services for psychosis. (Programme 3)

New practices were introduced one at a time, in agreement with managers and case managers, in a way that respected the adaptability of every clinician. (Programme 6)

[The] new manager and co‐manager do not share the same priorities for change […]. The lead psychiatrist is experienced with good leadership and great influence at the management level. (Programme 13)

[The manager] lacks knowledge of the Cadre, gives contradictory directives to the case managers. (Programme 13)

Constant change of managers [makes] significant changes impossible. (Programme 16)

## 4 Discussion

This study identified eight themes that grouped factors influencing EIP implementation in a publicly funded healthcare system through deductive and inductive analysis of qualitative data. Most themes and subthemes revealed themselves as potential barriers or facilitators. The work environment, quality of physical premises, the capacity to offer interventions tailored to individual needs by having appropriate numbers and diversity of staff, and management's adherence to early intervention services' tenets were more clearly perceived as positively influencing the process of implementing and operating EIP.

As evidenced by our study's results, almost every theme and subtheme contained statements suggesting they might act as a barrier or a facilitator to programme implementation, revealing the themes' multiple facets. This multidimensionality has been highlighted in other studies, including a systematic review of barriers and facilitators in EIP implementation (O'Connell et al. 2021; Reed et al. 2025).

This study was conducted 2–3 years after the government provided additional dedicated funding and implementation support to EIP across Quebec, with specific funding allocated to developing new programmes. In our previous study on the same programmes, we found that such additional support helped improve adherence to core components of the EIP model and allowed newer programmes to rapidly implement high‐quality services (Bertulies‐Esposito et al. 2022). Despite this, programmes implemented after these policy changes did not express more positive views on implementation processes and potential modulators than the more established ones which did not benefit from such support during their early implementation phase. In fact, programmes implemented prior to these policy changes had more positive perceptions in some subthemes, including the quantity and quality of physical resources, and the ability to provide tailored interventions to service users. These findings highlight the complex nature of human systems and processes in EIP, and that implementation challenges may have been perceived differently by respondents according to their experience in EIP. The additional governmental support was associated with the publication of local guidelines and standards for EIP, a model that greatly differs from standard outpatient psychiatric care in most settings. Programme leaders in newer programmes may have also had to adapt their practice to adhere to the EIP model of care, resulting in more critical perspectives of the implementation process.

### 4.1 System‐Related Factors

Funding and related issues, such as clinical and administrative staffing, and adequacy of physical resources, were the most often cited factors influencing the implementation of EIP, in line with results from a recent systematic review (Darker et al. 2023; Hallett et al. 2024; Mancini et al. 2009; O'Connell et al. 2021; Whitley et al. 2009). This underfunding has been associated with a lack of training, insufficient staffing and lack of outreach and community interventions, with probable impacts on programme fidelity and possibly outcomes. Several programme leaders were not only aware and concerned about immediate funding but also questioned the sustainability of their financial resources. Another intricately linked factor was the state of physical resources and their impact on programme implementation: many programmes cited a lack of offices to meet with youth and shared offices, which add a layer of complexity to planning day‐to‐day activities, especially when administrative support is lacking.

Our findings highlighted the benefits of organisational support and trust in EIP by management, which are key to securing adequate funding and durable implementation and programme operations, in line with other studies (Mancini et al. 2009; O'Connell et al. 2021). This suggests that funding and management buy‐in to EIP are cornerstones of successful programme implementation, as these overarching organisational factors affect directly and indirectly the other barriers and facilitators identified in this study.

### 4.2 Service‐Related Factors

Staff workload and high caseloads were associated with delays in service delivery and limited time for outreach, hindering early detection and intervention, which are foundational to this model (Addington et al. 2013; Hegelstad et al. 2012). This work overload can also impede efforts to offer diverse interventions (e.g., outreach activities), as recommended by provincial (Ministère de la santé et des services sociaux 2022) and international guidelines (Early Psychosis Guidelines Writing Group and EPPIC National Support Program 2016; Ehmann et al. 2010; National Institute for Health and Care Excellence 2016). Furthermore, the impact of high caseloads on staff turnover in mental health services has been documented (Hallett et al. 2024), which in turn increases the workload of remaining clinicians, possibly hindering their well‐being while also adding the burden of recruiting and training new staff. In turn, this increased workload resulted in programmes ending episodes of care and creating waiting lists, which have been reported in other studies on EIP implementation, although these responses are in total contradiction to the EIP model of care (Lester et al. 2009). Ultimately, this cycle can affect programme fidelity, implementation outcomes, and quality of care, as noted by some respondents in this study. Notably, outreach interventions are often sacrificed to compensate for inadequate staffing and high caseloads.

Nevertheless, team morale and workspace atmosphere were mostly cited as facilitators to programme implementation. Indeed, EIP is often composed of motivated and enthusiastic clinicians, which can improve individual and team resilience despite multi‐level stressors, including high‐complexity caseloads, limited physical, technological, financial resources and training opportunities. This could be attributed to clinicians finding a sense of purpose in their practice and support from their colleagues. Strategies to nourish team morale should be encouraged, such as empowering clinicians' initiatives which are in line with best practices, improved clinical supervision opportunities, promoting common team training and social activities, and underlining patients and families' satisfaction or the team successes by management teams could be some examples (Hyrkäs 2005; Johnson et al. 2018).

### 4.3 Staff‐Related Factors

Closely linked to the two previous categories, several staff‐related factors were identified, including knowledge and adoption of guidelines, impact of turnover and training and clinical supervision. As mentioned previously, programme fidelity can be affected by a myriad of factors, but it appears critical that guidelines and standards should be mastered by senior clinical staff and management. This was brought up by several respondents, both as a barrier to implementation and as a facilitator. It seems that when underfunding and understaffing are rampant in EIP, addressing emergent clinical needs is prioritised over understanding the spirit and the letter of guidelines. However, not following guidelines can impact fidelity, which could decrease intervention effectiveness (Addington et al. 2018) and further increase pressure on clinicians. Moreover, with several respondents reporting the positive impacts of clinicians' strong belief in EIP on team morale and cohesion, burnout and turnover rates might ultimately increase if staff are not guided by guidelines.

Providing adequate training and clinical supervision to clinicians is paramount in offering high‐quality and high‐fidelity intervention and was reported as an area of urgent need by several respondents. Furthermore, ongoing training and supervision can improve staff retention (Hallett et al. 2024) and decrease risks of burnout (Hyrkäs 2005), underscoring the possibility of multifaceted gains for EIP implementation when training is valued and offered consistently.

### 4.4 Strengths

All EIP in Quebec were contacted for this survey, and 85% of programmes completed it, including programmes in urban, suburban, and rural areas. This study provides insight into factors influencing implementation of EIP in a variety of geographical and sociodemographic settings. This study is the first to assess factors influencing implementation of EIP in Quebec, and offers further details of implementation experience by clinicians and team leaders, going beyond standards of care and service delivery, which have been reported elsewhere (Bertulies‐Esposito et al. 2022).

### 4.5 Limitations

The survey was completed by clinical team leaders and lead psychiatrists, which limits the diversity of perspectives that were collected in this survey. Future research should involve a greater breadth of stakeholders, including service users and their families, clinicians and management staff, and the use of focus groups or in‐depth individual interviews could allow a deeper understanding of the discussed topics.

As this study was conducted in an entirely publicly funded healthcare system in a high‐income country, results might not be generalisable to all settings. However, as noted previously, our results are in line with previous findings in mental healthcare implementation literature in high‐income settings, suggesting that common factors are present in a variety of settings, highlighting the need for ongoing implementation research in EIP. We elected to conduct an inductive data analysis instead of employing an implementation science framework for this study. The latter could have led to different results, but the former allowed us to be guided by the data. Another specific factor that could have influenced this study's results is that the provincial government had recently announced a host of measures to promote and support widespread EIP implementation (Bertulies‐Esposito et al. 2022). Nevertheless, underfunding, subsequent understaffing, and difficulties with local management were noted across programmes, suggesting that there is a gap between announced institutional support and the reality of local programmes in decentralised healthcare organisations. Moreover, it is likely that the service implementation issues raised here are even more crucial in systems in which there are no EIP‐specific guidelines and funding.

## 5 Conclusion

Implementation of EIP can be affected by multiple factors at the system, service and staff levels. Our findings underline the importance of assessing these factors continuously during implementation, as these can also impact clinical services and outcomes. Considering resource constraints in healthcare systems, efficient assessment mechanisms should be developed. For instance, learning health systems could provide rapid and automated feedback to inform programme leaders and managers on strategies to maximise potential for implementation success. Future research on implementation processes should include multiple stakeholders' views, thereby identifying and monitoring a broader, inclusive range of factors that may influence implementation processes. Given the diverse nature of factors identified in this study, careful prioritisation of areas of need adapted to local realities is needed for effective implementation of EIP.

## Data Availability

The data that support the findings of this study are available from the corresponding author upon reasonable request.
